# Clinical and Genetic Risk Factors for Drug-Induced Liver Injury Associated with Anti-Tuberculosis Treatment—A Study from Patients of Portuguese Health Centers

**DOI:** 10.3390/jpm12050790

**Published:** 2022-05-13

**Authors:** Maria João Cavaco, Celeste Alcobia, Bárbara Oliveiros, Luís Alcides Mesquita, Aurora Carvalho, Fernando Matos, José Miguel Carvalho, Miguel Villar, Raquel Duarte, João Mendes, Carolina Ribeiro, Carlos Robalo Cordeiro, Fernando Regateiro, Henriqueta Coimbra Silva

**Affiliations:** 1 Oeste Hospital Center, 2560-295 Lisboa, Portugal; 2 Department of Pneumology, Coimbra Hospital and Universitary Centre, 3004-561 Coimbra, Portugal; mcalcobia@hotmail.com (C.A.); carlos.crobalo@gmail.com (C.R.C.); 3 Pneumological Diagnostic Center of the Centre, 3000-075 Coimbra, Portugal; 4 Laboratory of Biostatistics and Medical Informatics, Faculty of Medicine, University of Coimbra, 3000-548 Coimbra, Portugal; boliveiros@fmed.uc.pt; 5 Coimbra Institute for Clinical and Biomedical Research (iCBR), Faculty of Medicine, University of Coimbra, 3000-548 Coimbra, Portugal; hsilva@fmed.uc.pt; 6 Institute of Medical Genetics/UC Genomics, Faculty of Medicine, University of Coimbra, 3000-548 Coimbra, Portugal; alcidesn@ci.uc.pt (L.A.M.); jmendes@uc.pt (J.M.); carolina_ribeiro86@hotmail.com (C.R.); fregateiro@gmail.com (F.R.); 7 Department of Pneumology, Vila Nova de Gaia Hospitalar Centre, 4434-502 Vila Nova de Gaia, Portugal; auroracarvalho.gaia@gmail.com; 8 Pneumological Diagnostic Center of Aveiro, 3810-042 Aveiro, Portugal; fernandonoronhamatos@gmail.com; 9 Pneumological Diagnostic Center of Santarém, 2005-324 Santarém, Portugal; zemig51@gmail.com; 10 Pneumological Diagnostic Center of Venda Nova, 2700-220 Lisboa, Portugal; mtvillar@mail.telepac.pt; 11 Pneumological Diagnostic Center of Vila Nova de Gaia, 4400-088 Vila Nova de Gaia, Portugal; raquelafduarte@gmail.com

**Keywords:** tuberculosis, DILI, NAT2, ABCB11, isoniazid, RUCAM

## Abstract

Drug-induced liver injury (DILI) is an unpredictable and feared side effect of antituberculosis treatment (AT). The present study aimed to identify clinical and genetic variables associated with susceptibility to AT-associated hepatotoxicity in patients with pulmonary tuberculosis treated with a standard protocol. Of 233 patients enrolled, 90% prospectively, 103 developed liver injury: 37 with mild and 66 with severe phenotype (DILI). All patients with mild hepatitis had a RUCAM score ≥4 and all patients with DILI had a RUCAM score ≥ 6. Eight clinical variables and variants in six candidate genes were assessed. A logistic multivariate regression analysis identified four risk factors for AT-DILI: age ≥ 55 years (OR:3.67; 95% CI:1.82–7.41; *p* < 0.001), concomitant medication with other hepatotoxic drugs (OR:2.54; 95% CI:1.23–5.26; *p* = 0.012), *NAT2* slow acetylator status (OR:2.46; 95% CI:1.25–4.84; *p* = 0.009), and carriers of p.Val444Ala variant for *ABCB11* gene (OR:2.06; 95%CI:1.02–4.17; *p* = 0.044). The statistical model explains 24.9% of the susceptibility to AT-DILI, with an 8.9 times difference between patients in the highest and in the lowest quartiles of risk scores. This study sustains the complex architecture of AT-DILI. Prospective studies should evaluate the benefit of *NAT2* and *ABCB11* genotyping in AT personalization, particularly in patients over 55 years.

## 1. Introduction

In the 21st century, a quarter of the world’s population is still infected with Mycobacterium tuberculosis and thus at risk of developing active tuberculosis (TB) during their lifetime [1]. Standard antituberculosis treatment (AT) [2] is associated hepatotoxicity in 1–36% of treated patients. The spectrum of presentation is broad, ranging from asymptomatic and frequently transient elevations of liver aminotransferases in 20% of patients, to more severe phenotypes imposing interruption and modification of treatment (currently classified as drug-induced liver injury (DILI)) and even acute liver failure in 1–5% of cases [3]. Despite its high efficacy, isoniazid (INH) is the most frequently associated drug [4], though rifampicin (RIF) and pyrazinamide (PZ) have also been implicated, with the combination of drugs showing an increased risk [5,6].

AT-associated hepatotoxicity is a complex phenotype involving multiple low penetrance genetic and non-genetic variables [7]. The development of integrated risk models to assess individual susceptibility may lead to less adverse reactions, better adherence to therapy, and lower risk of emergence of resistant forms. Age, female gender, race, alcoholism, and pre-existing liver disease are some of the risk factors thought to be involved, although studies show contradictory results [3,8]. The characterization of single nucleotide polymorphisms (SNP) from the gene encoding arylamine N-acetyltransferase 2 (NAT2) enzyme, responsible for 80% of INH’s clearance, allows to classify individuals as slow (SA), intermediate (IA), or rapid acetylators (RA) [9,10]. According to most studies and meta-analyses, SA have an increased susceptibility to the development of INH-induced hepatotoxicity [7,9,10,11,12,13,14,15,16].

The cytochrome P450 2E1 (CYP2E1) is a phase I metabolizing enzyme that converts INH subproducts hydrazine and acetyl hydrazine into reactive hepatotoxic metabolites [17]. The SNP rs2031920, a 2Kb gene upstream variant, has been associated with higher enzymatic activity [18,19]. Glutathione S-Transferases (GSTs) are phase II metabolizing enzymes with an important role in the detoxification of metabolites resulting from the biotransformation of xenobiotics by phase I enzymes [17]. Copy number variations (CNV) consisting in the deletion of Glutathione S-Transferase theta 1 (*GSTT1*) and Glutathione S-Transferase Mu 1 (*GSTM1*) have been associated with AT-DILI, although with controversial results [20,21].

Loss of function variants in the gene ATP-binding cassette, subfamily B, member 11 (*ABCB11*), encoding the bile salt export pump (BSEP), may also be implicated. The SNP rs2287622 (p.Val444Ala) has been related to an increased risk of intrahepatic cholestasis in different clinical situations, including tuberculosis treatment [22]. Moreover, an in vitro study proved that the association of INH with RFP significantly down-regulated the expression of BSEP in liver extracts of mice [23].

IL6 is a pleiotropic cytokine with a complex role, including pro-inflammatory, anti-inflammatory, and regenerative responses [24]. In the liver, IL6 is a major inducer of the acute phase response after infection, simultaneously improving hepatic regeneration and repair, but in more chronic exposure, it can actually contribute to liver damage [25,26,27]. *IL6* gene upstream SNPs rs1800796 and rs1800797 have been associated with susceptibility to liver diseases though the impact and risk genotypes varied with ethnicity [28,29]. Although NAT2 function is specifically related to INH-DILI, CYP2E1, GSTs, BSEP, and IL6 have broad-range roles and may also be implicated in the hepatotoxic effect of other antituberculosis drugs.

The aim of the present study was to identify genetic and clinical variables associated with susceptibility to AT-associated hepatotoxicity in patients with pulmonary disease.

## 2. Materials and Methods

### 2.1. Patients

A total of 233 unrelated patients diagnosed with pulmonary tuberculosis between 2004 and 2017 were studied. From 2004 to 2008, the study was retrospective and recruited patients (10%) from Coimbra Hospital and University Centre (CHUC) and from the Pneumological Diagnostic Center (CPD) of Coimbra. From 2008, the study was prospective and also included patients from the CPDs of Vila Nova de Gaia, Aveiro, Venda Nova, and Santarém.

Patients were classified as “cases” if they developed AT-associated hepatotoxicity or as “controls” if they did not. For patients that agreed to participate, a blood sample was collected for genetic analysis and a specific clinical record was created. Since 2008, for “cases” genotyping of *NAT2* was immediately performed to personalize INH dosing. To limit the number of controls, for every patient becoming a case, the next patient or the next two patients were included as controls, thus assuring a blind selection. Genetic studies other than *NAT2* for cases and all genes for controls were only performed after all patients had been enrolled, and only in the selected individuals. Overall, of the initially selected patients, 10% refused to participate and six patients were posteriorly excluded: three had incomplete genotype results, two had hepatotoxicity attributed to other causes, and one patient developed cancer.

Blood samples were referred by code number. All participants were informed about the study and signed an informed consent. The study was approved by the Ethics Committee of Coimbra Hospital and University Centre (Approval Code: CES011, Approval Date: 15 January 2008).

Eligibility criteria: Diagnosis of pulmonary tuberculosis, no contraindication to the standard treatment, age >16 years old, normal baseline laboratory tests including: aspartate aminotransferase (AST), alanine aminotransferase (ALT), alkaline phosphatase (ALP), total bilirubin, and γ glutamyl transpeptidase. Human hepatitis virus (A, B and C) and HIV serology should be negative.

Exclusion criteria: pregnancy, intellectual disability, incomplete data or genotyping results; hepatitis attributed to other causes than AT drugs by causality assessment applying Roussel Uclaf Causality Assessment Method (RUCAM) [30], development of severe co-morbidity during follow-up, previous diagnosis of hepatitis of any cause, and evidence of non-compliance.

World Health Organization (WHO) guidelines for Tuberculosis [2,31,32] were followed for the management of patients, including initial standard treatment with 2 months of isoniazid (INH), rifampin (RIF), pyrazinamide (PZA), and ethambutol (EMB) followed by a continuation phase of 4 months of INH and RIF. With the exceptions of patients assuring a high compliance, directly observed treatment was adopted. Patients had a first follow up appointment 15 days after initiating therapy and monthly thereafter. In each appointment, a clinical record was fulfilled, and blood levels of liver enzymes were evaluated.

AT-associated hepatotoxicity was classified in three grades: (1) mild hepatitis, considering an elevation of serum concentration of ALT exceeding two-fold the upper limit of normal (ULN) [33]; (2) severe/DILI, considering an ALT level of more than five-fold above the ULN, or an ALP level of more than two-fold above the ULN; or ALT level of more than three-fold above the ULN, with the simultaneous elevation of total bilirubin levels to more than two-fold above the ULN [34]; (3) liver failure, considering a grade 2 criteria plus death or liver transplantation. The RUCAM score [30] was used for the adjudication of patients as having AT-associated hepatotoxicity (defined as cases). The liver injury pattern was assessed by the R value [33]. The earliest identified pattern of liver injury was recorded.

Co-morbidities at the time of diagnosis excluded transient acute diseases. Active smoking habits are described as: “not present” (less than 1 cigarette a day in the last month) or “present” [35]. Alcoholic intake refers to average habits in the last three months preceding the diagnosis of tuberculosis. Patients were classified as having “no alcohol” consumption if drinking no more than 20 g of pure alcohol a day for men or half that amount for women [36]. During treatment, patients were instructed to reduce their alcohol drinking habits by at least 50%. Concomitant chronic unspecified medication and concomitant chronic medication, including hepatotoxic drugs, were registered. Drugs were classified as hepatotoxic according to EASL guidelines [37]. These drugs were excluded as the cause of liver injury (RUCAM score < 3).

### 2.2. Genotyping

Extraction of DNA was performed from peripheral blood samples using the “NZY Blood gDNA Isolation” kit.

For the *NAT2* gene, the gold standard 11 SNPs were evaluated by Sanger sequencing [38,39]. The SNPs rs2287622 from *ABCB11* gene, rs2031920 form *CYP2E1* gene, and rs1800796 and rs1800797 from *IL6* gene were also characterized by Sanger sequencing. To identify *GSTT1* and *GSTM1* deletions, a multiplex PCR reaction was performed using a sequence of the β-globin gene as internal control [40]. The evidence of amplification, described as positive, identifies the presence of at least one allele, not distinguishing homozygotes from heterozygotes, while the absence of amplification, described as null, identifies homozygosity for the deletions.

Primers and annealing temperatures are described in Supplementary Table S1.

### 2.3. Statistical Analysis

Statistical analysis was performed with the software SPSS Statistics, Version 25 (SPSS, Inc., IBM Company, Armonk, NY, USA). Hardy–Weinberg equilibrium was tested applying a chi-squared test. A *p*-value below 0.05 was considered statistically significant. Univariate analyses were performed for each risk factor. For age, the Mann–Whitney test was used. To transform age in a binary variable, ROC curve analysis was performed (cut-off value for hepatotoxicity: ≥55 years, sensitivity 56.2%, specificity 73.8%). Given its normal distribution, weight was assessed with an ANOVA test. The remaining variables were analyzed using Fisher’s exact test. Multicollinearity was determined between independent variables using Fisher’s exact test. The identification of predictors of hepatotoxicity was performed through logistic regression. The adequacy of the logistic regression models was evaluated by the Hosmer–Lemeshow test and Nagelkerke R2 was computed as a measure of the variance explained by the model. Quartile analysis of cases and controls based on the range of probability values obtained from the multivariate regression model was also performed.

## 3. Results

### 3.1. Patients Characteristics and Univariate Analysis

Of the 233 patients included in the study, 103 (44.2%) developed AT-associated hepatotoxicity (cases): 37 patients were classified as grade 1, 61 patients as grade 2, and five patients as grade 3. Patients with grade 1 (36% of hepatotoxicity group) were described as having mild hepatotoxicity and grade 2 and three patients (64% of hepatotoxicity group) were joined and described as severe hepatotoxicity/DILI. RUCAM scores for causality assessment of AT drugs were as follows: all patients in grade 2 had a score ≥ 6 (probable), with 14 patients (23%) reaching a score ≥ 8; all patients with grade 1 had a score ≥ 4 (possible), 9 (24%) reaching a score ≥ 6.

The hepatocellular pattern was the most common among patients with severe hepatotoxicity/DILI (Table 1). For patients with mild forms, there was a greater heterogeneity, with the mixed pattern being the more frequent.

Clinical characteristics evaluated for cases and controls are described in Table 2.

A univariate analysis was also performed for patients with mild hepatitis and AT-DILI and described in Supplementary Tables S2 and S3.

For alcohol consumption, other medication, and other hepatotoxic drugs, different subgroups were analyzed but only results with statistical significance are described in Table 2. A total of 116 patients (49.8%) had no concomitant medication. The most common hepatotoxic drugs registered were statins and oral contraceptives.

*NAT2* genotypes were grouped according to acetylator phenotype (Table 3): 126 patients (54.1%) were SA, 95 (40.8%) were IA, and 12 patients (5.1%) were RA. The genotypic and phenotypic frequencies found in this study were similar to those previously described for the Portuguese population [40,41]. For other analyzed genes, the frequencies of genotypes in the global sample and in patients with and without hepatotoxicity are also described in Table 4. Hardy–Weinberg equilibrium was confirmed for all SNPs (*p* > 0.05).

For each gene, all other combinations of SNP variants were tested but no statistically significant association was revealed.

Analysis of ethnicity interference showed that the genotype AG of *IL6* rs1800797 SNP and presence of at least one allele of *GSTT1* are more frequent among Caucasians (*p* = 0.002 and *p* = 0.036, respectively, results not shown). However, in a logistic regression analysis involving the interaction between each of these genetic variants and race, none was found to interfere (Race and *IL6*: OR:1.28, *p* = 0.692; Race and *GSTT1* OR:0.78, *p* = 0.668). Thus, the inclusion of non-Caucasians does not introduce a bias in the results.

### 3.2. Multivariate Analysis

A logistic multivariate analysis for all cases (Table 4) was performed, including variables with a *p* < 0.05 in univariate analysis (Table 2 and Table 3). The same analysis was also performed for DILI cases compared to controls (Table 4).

Finally, a refined analysis (Table 5) was performed including only variables with statistical significance in the previous logistic multivariate analysis.

Each variable increases the susceptibility to AT-associated hepatotoxicity by 2–3 times. For all cases, the presented model explains 24.2% of the susceptibility to AT-associated hepatotoxicity. Considering the refined logistic regression model, the achieved Nagelkerke R Square (0.242), and the four predictors identified at a 5% significance level with a sample of 233 subjects, the attained statistical power for the analysis is 87.2%.

For patients with severe hepatotoxicity/DILI (Table 5), the same variables were identified: age ≥ 55 years (OR:3.67; 95% CI:1.82–7.41; *p* < 0.001), concomitant medication with other hepatotoxic drugs (OR:2.54; 95% CI:1.23–5.26; *p* = 0.012), SA status (OR:2.46; 95% CI:1.25–4.84; *p* = 0.009) and presence of CC genotype for *ABCB11* SNP (OR:2.06; 95% CI:1.02–4.17; *p* = 0.044). The statistical model also explains about 25% of the susceptibility to this more severe phenotype.

A logistic multivariate analysis was also performed for patients with mild hepatotoxicity (Supplementary Table S4). The presence of other hepatotoxic drugs and SA status were the only risk factors identified (*p* = 0.025 and *p* = 0.038, respectively), with age ≥ 55 years showing an almost statistically significant effect (*p* = 0.075).

### 3.3. Quartile Analysis of Risk Scores

**Figure 1:**
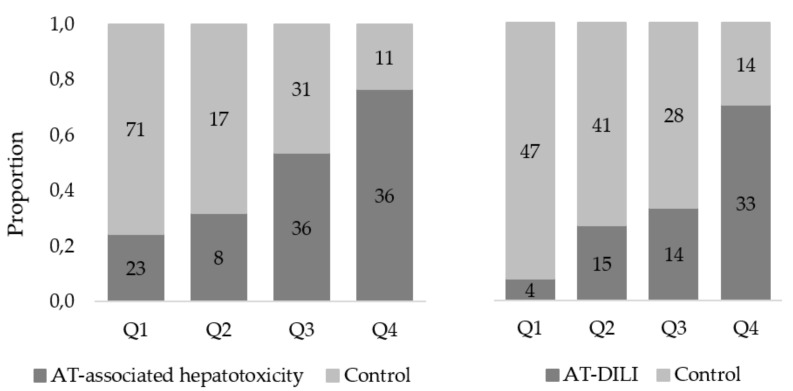
Proportion of cases and controls in each quartile using the best predictive model for AT-associated hepatotoxicity and AT-DILI, respectively. The dark bars represent the proportion of cases which fall into each quartile and the light bars represent the proportion of controls. The number of cases and controls who fall within each quartile are indicted in each block.

Quartile analysis of risk scores showed a 3.5 times difference on susceptibility to AT-associated hepatotoxicity (both forms) between patients in the highest and the lowest quartile (Figure 1).

For patients with severe forms, AT-DILI, there is an 8.9 times difference between patients in the highest and in the lowest quartiles of risk scores (Figure 1).

## 4. Discussion

Despite extensive research efforts, the mechanisms beyond AT-associated hepatotoxicity are still not fully understood. In this study, we searched for genetic and non-genetic variants associated with this complex phenotype. Regarding non-genetic risk factors, results show that age ≥ 55 years was associated with a triple increase in the risk of AT-associated hepatotoxicity. Other studies confirm that older age is a risk factor [42] though the mechanisms involved are unclear [43]. Aging is known to associate with the deterioration of renal function and with a decrease of organ volume, blood flow, and cytochrome-mediated metabolism in the liver, all of which might affect drug pharmacokinetics [44]. Despite these changes, renal and liver function are essentially preserved in healthy older humans. It is also important to consider factors, such immunoinflammatory modifications [45] and polypharmacy, which are more common among the elderly [44]. In our patients, chronic diseases and concomitant medication were not risk factors, but the use of other hepatotoxic drugs increased the risk increased the risk by 2.3 times. Given this association, in clinical practice, the suspension of other hepatotoxic medication before starting AT treatment should be evaluated on a case-by-case basis. Contrarily to other authors, gender was not identified as a risk factor [44]. Pre-existing liver disease was not evaluated in this study as patients without normal baseline laboratory tests were excluded. In multivariate analysis, alcohol intake and smoking habits were not identified as risk factors. These are subjective variables as they depend on the reliability of patients’ responses. Though alcohol consumption is frequently referred to as a risk factor and is included in RUCAM score [30], a recent study failed to establish an association with DILI attributed to isoniazid [8]. The fact that no patient with baseline evidence of liver damage was included and that alcohol intake was restricted during treatment may also explain our results. The interference of all these clinical factors in DILI remains a subject of discussion [37].

Our results confirm the role of *NAT2* SA genotypes in AT-DILI and show that the impact is extendable to mild forms of AT-associated hepatotoxicity. There is evidence that genotyping-based INH dosing may significantly decrease the incidence of AT-associated hepatotoxicity and early treatment failure, allowing a 31% reduction in absolute risk of unfavorable events [16]. It was also suggested that patients with SA status would likely benefit from closer surveillance [14,15]. Within the SA group, there is heterogeneity in phenotype due to variations in enzyme activity conferred by different alleles, with some alleles associating with “ultra-slow” acetylation, supporting the advantage of *NAT2* genotyping [11]. *NAT2* genotyping was performed with the 11-SNP panel, considered to be the gold standard method, but more practical approaches of seven- and four-SNP panels show a classification accuracy of 98.4% in USA individuals, including 79.3% Caucasian, 10.55% African-American, and 7.03% Hispanic [39]. We verified that the seven-SNP panel could classify all our patients. Genotype ambiguities were within the same acetylator activity group and did not compromise phenotype classification.

*ABCB11* gene SNP rs2287622 also shows impact in AT-DILI but not in mild AT-associated hepatotoxicity. This gene has been implicated in hereditary and acquired forms of liver cholestasis but population studies on the association with AT-DILI are scarce [22]. In our patients, even though the most common pattern was the hepatocellular, the presence of CC genotype doubled the chance of AT-DILI. For the functional genetic polymorphisms studied in *CYP2E1, GSTM1, GSTT1,* and *IL6* genes, no association was found. For these genes, conflicting results have been published. Considering recent meta-analyses, two support the roles of *CYP2E1, GSTM1,* and *GSTT1* in AT-DILI [19,20], one does not implicate *GSTT1* [21], and in another *CYP2E1* could only be implicated in East Asian populations [18]. In a trans-ethnic meta-analysis from Nicoletti et al., *CYP2E1* was not associated with AT-DILI [46]. For *IL6* association with AT-DILI, the few results reported are not conclusive [29].

Variation in population genetic background and in environmental exposures, namely to herbals, regional medication policies, and prescription habits, may account for some discrepancies in studies evaluating susceptibility to AT-DILI [44]. Differences in study design, including genotyping methodologies and criteria for AT-DILI, may also contribute. AT-DILI is a complex phenotype better described by multifactorial models, including multiple genetic and non-genetic factors, but most studies do not analyze the impact of variables, such as pre-existing liver diseases or concomitant medication, though not excluding them, as is the case of meta-analyses and studies well designed to explore the association only with genetic variants [46]. The impact of the identified risk factors in the predictability of the pathologic condition is also frequently not referred to.

In this study, when mild and more severe liver injury were separately evaluated, different risk profiles emerged. For mild presentations, characterized by a more heterogeneous hepatitis pattern, including a higher frequency of cholestatic forms, only age ≥ 55 years, and *NAT2* SA status, could be implicated. This suggests that mild, transient forms and more severe phenotypes are related and share some risk factors.

For AT-DILI, quartile analysis of risk scores shows an 8.9 times difference between patients in the highest and lowest quartiles. Yet, exploring seven genetic and nine clinical variables, only 25% of the risk of AT-DILI could be predicted, supporting the multifactorial and polygenic architecture of the phenotype. In contrast, INH blood levels behave as a quasi-monogenic trait, mostly depending on *NAT2* genotype. A role for immune mediated response in AT-DILI events has been suggested [4] and is supported by a recent association described with HLA-B*52:01 [46].

This study has several limitations. First, as all hepatotoxicity events occurred when patients were submitted to treatment with four drugs, although INH is thought to be the most frequently involved, no specific antituberculosis drug can be implicated. In order to assess INH-DILI susceptibility factors, studies, including genotyping and monitoring of plasma INH toxic metabolites, should be performed on individuals treated with isoniazid alone, as in latent TB [47]. Second, to increase statistical power and to enable variant-interaction analysis, the sample size should be extended. The sample dimension also precluded a genome wide association approach. Information on alcoholic and smoking habits and therapeutic compliance was self-reported and may not be accurate. The possible role of epigenetic factors was not evaluated [48,49].

## 5. Conclusions

In this study, exploring multiple genetic and clinical variables, four risk factors for AT-DILI were identified: *NAT2* SNPs determining SA phenotype, CC genotype for *ABCB11* SNP rs2287622, age ≥ 55 years, and concomitant use of hepatotoxic drugs. The overall effect of the studied variables is modest, which suggests a complex interaction of many still unknown genetic, epigenetic, and environmental factors. For clinical translation, prospective studies should evaluate the role of *NAT2* and *ABCB11* genotyping in the AT personalization, particularly in patients over 55 years. Due consideration should also be given to the interruption of other hepatotoxic medication during AT treatment.

## Figures and Tables

**Table 1:** Distribution of hepatitis pattern according to the grade of hepatotoxicity.

Hepatitis Pattern	Grade of Hepatotoxicity		
	Mild *n* (%)	DILI *n* (%)	Total *n* (%)
Hepatocellular	10 (33.3%)	60 (90.9%)	70 (72.9%)
Cholestatic	7 (23.3%)	4 (6.1%)	11 (11.5%)
Mixed	13 (43.4%)	2 (3.0%)	15 (15.6%)
Total	30 (100%)	66 (100%)	96 (100%)

*n*—number of patients.

**Table 2:** Characterization of non-genetic risk factors.

Clinical Variables	Controls *n* (%)	Cases *n* (%)	Global *n* (%)	*p*	OR (95% IC)
**Age** (17–87 years)					
<55 years	96 (73.8%)	46 (44.7%)	142 (60.9%)	**<0.001**	
≥55 years	34 (26.2%)	57 (55.3%)	91 (39.1%)		3.49 (2.02–6.07)
Mean (sd)	45.1 (16.1)	55.2 (19.0)	49.6 (18.1)		
**Gender**					
Female	39 (30.0%)	36 (35.0%)	7 (32.2%)	0.422	1.25 (0.72–2.18)
Male	91 (70.0%)	67 (65.0%)	158 (67.8%)		
**Race**					
Caucasian	120 (92.3%)	95 (92.42%)	215 (92.3%)	0.983	
Non-Caucasian	10 (7.7%)	8 (7.8%)	18 (7.7%)		1.01 (0.38–2.66)
**Weight** (33–103 kg)					
Mean (sd)	61.5 (11.3)	62.2 (11.8)	61.8 (11.5)	0.419	
**Chronic Diseases**					
No	63 (48.5%)	36 (35.0%)	99 (42.5%)	**0.039**	
Yes	67 (51.5%)	67 (65.0%)	134 (57.5%)		1.75 (1.03–2.98)
**Smoking Habits**					
No	79 (60.8%)	82 (79.6%)	161 (69.1%)	**0.002**	
Yes	51 (39.2%)	21 (20.4%)	72 (30.9%)		0.39 (0.22–0.72)
**Alcohol Consumption**					
No	85 (65.4%)	82 (79.6%)	167 (71.7%)	**0.018**	
Yes	45 (34.6%)	21 (20.4%)	66 (28.3%)		0.48 (0.26–0.88)
**Other Medication**					
<3 drugs	114 (87.7%)	76 (73.8%)	190 (81.5%)	**0.008**	
≥3 drugs	16 (12.3%)	29 (26.2%)	43 (18.5%)		2.53 (1.28–5.01)
**Other Hepatotoxic Drugs**					
No	106 (81.5%)	57 (55.3%)	163 (70.0%)	**<0.001**	
Yes	24 (18.5%)	46 (44.7%)	70 (30.0%)		3.56 (1.98–6.42)

*n*—number of patients; *p*—*p* value; OR—odds ratio; 95% IC—confidence interval; sd—standard deviation. Statistically significant results are highlighted in bold.

**Table 3:** Characterization of genetic risk factors.

Genotypes	Controls *n* (%)	Cases *n* (%)	Global *n* (%)	*p*	OR (95% IC)
***NAT2*** **/Acetylation status**					
SA	58 (44.6%)	68 (66.0%)	126 (54.1%)		
IA	65 (50.0%)	30 (29.1%)	95 (40.8%)	0.001	0.39 (0.23–0.69)
RA	7 (5.4%)	5 (4.9%)	12 (5.1%)	0.418	0.61 (0.18–2.02)
RA + IA vs. SA	72 (55.4%)	35 (34.0%)	107 (45.9%)	**0.001**	0.42 (0.24–0.71)
***ABCB11***					
TT + TC	21 + 76 (74.6%)	13 + 48 (59.2%)	158 (67.8%)	**0.013**	
CC	33 (25.4%)	42 (40.8%)	75 (32.2%)		2.024 (1.16–3.53)
***GSTM1***					
Null	69 (53.1%)	49 (47.6%)	118 (50.6%)	0.404	
Positive	61 (46.9%)	54 (52.4%)	115 (49.4%)		1.247 (0.74–2.09)
***GSTT1***					
Null	17 (13.1%)	9 (8.7%)	26 (11.2%)	0.299	
Positive	113 (85.9%)	94 (91.3%)	207 (88.8%)		1.571 (0.67–3.69)
***IL6* rs1800797**					
AA + AG	16 + 54 (53.8%)	8 + 54 (60.2%)	132 (56.7%)	0.332	
GG	60 (46.2%)	41 (39.8%)	101 (43.3%)		0.772 (0.46–1.30)
***IL6* rs1800796**					
CC + CG	2 + 16 (13.8%)	1 + 17 (17.5%)	36 (15.5%)	0.447	
GG	112 (86.2%)	85 (82.5%)	197 (84.5%)		0.759 (0.37–1.55)
***CYP2E1***					
CC	114 (87.7%)	93 (90.3%)	207 (88.8%)	0.532	
TT + CT	1 + 15 (12.3%)	0 + 10 (9.7%)	26 (11.2%)		1.305 (0.57–3.01)

*n*—number of patients; *p*—*p* value; OR—odds ratio; 95% IC—confidence interval; sd—standard deviation. Statistically significant results are highlighted in bold.

**Table 4:** Results of logistic multivariate analysis for all cases and DILI.

	All Cases vs. Controls			DILI Cases vs. Controls		
Variables	OR	IC 95%	*p*	OR	IC 95%	*p*
Age ≥ 55 years	**2.78**	**1.45–5.31**	**0.002**	**3.65**	**1.74–7.62**	**0.001**
Chronic Diseases	0.94	0.49–1.81	0.854	0.84	0.42–1.77	0.654
Alcohol intake	0.72	0.34–1.51	0.382	0.66	0.27–1.58	0.350
Smoking habits	0.61	0.29–1.25	0.175	0.66	0.29–1.51	0.325
Other medication ≥3 drugs	1.19	0.49–2.87	0.691	1.00	0.37–2.69	0.993
Other hepatotoxic drugs	**2.35**	**1.15–4.81**	**0.019**	**2.31**	**1.01–5.28**	**0.048**
*Slow Acetylator*	**2.52**	**1.39–4.57**	**0.002**	**2.55**	**1.28–5.07**	**0.008**
*ABCB11* *–* *CC*	**1.91**	**1.03–3.56**	**0.044**	1.96	0.96–4.00	0.064

OR—odds ratio; CI—confidence interval; *p*—*p* value. Nagelkerke R Square: 26.4%and 26.9%, respectively. Statistically significant results are highlighted in bold.

**Table 5:** Results of refined logistic multivariate analysis for all cases and DILI.

	All Cases vs. Controls			DILI Cases vs. Controls		
Variables	OR	IC 95%	*p*	OR	IC 95%	*p*
Age ≥ 55 years	**2.97**	**1.62–5.43**	**<0.001**	**3.67**	**1.82–7.41**	**<0.001**
Other hepatotoxic drugs	**2.74**	**1.44–5.21**	**0.002**	**2.54**	**1.23–5.26**	**0.012**
*Slow Acetylator*	**2.40**	**1.34–4.31**	**0.003**	**2.46**	**1.25–4.84**	**0.009**
*ABCB11-CC*	**1.98**	**1.07–3.67**	**0.030**	**2.06**	**1.02–4.17**	**0.044**

OR—odds ratio; CI—confidence interval; *p*—*p* value. Nagelkerke R Square: 24.2% and 24.9%, respectively. Statistically significant results are highlighted in bold.

## Supplementary Materials

The following supporting information can be downloaded at: https://www.mdpi.com/article/10.3390/jpm12050790/s1, Table S1: Primers used in amplification and sequencing reactions; Table S2: Characterization of non-genetic risk factors in mild hepatitis and DILI; Table S3: Characterization of genetic risk factors in mild hepatitis and DILI; Table S4: Results of logistic multivariate analysis for mild hepatitis.
