# Supplementary material for: Clinical and Genetic Risk Factors for Drug-Induced Liver Injury Associated with Anti-Tuberculosis Treatment—A Study from Patients of Portuguese Health Centers
Source: J Pers Med. 2022 May 13;12(5):790. doi: 10.3390/jpm12050790 (PMC9144180; doi:10.3390/jpm12050790)
Supplement: Supplementary file 1 [file jpm-12-00790-s001.zip › jpm-1663851-supplementary.pdf]

Supplement material

Supplement Table S1 – Primers used in amplification and sequencing reactions

Gene	Primer	Annealing temperature
NAT2	F: 5′ACACGAGGAAATCAAATGCTAAAG 3′<br>R: 5′CTGCCACATCTGGGAGGAG 3′	59ºC
	F: 5′GCTGGGTCTGGAAGCTCCTC 3′<br>R: 5′TTGGGTGATACATACACAAGGG 3′	59ºC
ABCB11	F: 5′ACACCGAGTATCAACACAAAGC 3′<br>R: 5′CCAGGACAGTCTCAATGTATGC 3′	56ºC
GSTM1	F: 5′GAACTCCCTGAAAAGCTAAAGC 3′<br>R: 5′GTTGGGCTCAAATATACGGTGG 3′	58ºC
GSTT1	F: 5′TTCCTTACTGGTCCTCACATCTC 3′<br>R: 5′TCACCGGATCATGGCCAGCA 3′	58ºC
HBB	F: 5′ CAACTTCATCCACGTTCACC 3′<br>R: 5′ GAAGAGCCAAGGACAGGTAC 3′	58ºC
*IL-6**	F: 5′ GGAGACGCCTTGAAGTAACTGC 3′<br>R: 5′ AGTTTCCTCTGACTCCATCGCAG 3′	56ºC
CYP2E1	F: 5′ CCAGTCGAGTCTACATTGTCA 3′<br>R: 5′ TTCATTCTGTCTTCTAACTGG 3′	60ºC

*Both SNPs are in the same amplicon

Supplement Table S2 - Characterization of non-genetic risk factors in mild hepatitis and DILI

Clinical Variables	Controls n (%)	Controls vs. Mild hepatitis			Controls vs. DILI		
		n (%)	*p*	OR (95% IC)	n (%)	*p*	OR (95% IC)
**Age**							
< 55 years	96 (73.8%)	19 (51.4%)	**0.006**		34 (40.9%)	**<0.001**	
≥ 55 years	34 (26.2%)	18 (48.6%)		2.82 (1.34-5.96)	39 (59.1%)		4.18 (2.24-7.82)
Mean (sd)	45.1 (16.1)	51.9 (18.9)			57.0 (17.9)		
**Gender**							
Female	39 (30%)	10 (27.0%)	0.901		26 (39.4%)	0.148	
Male	91 (70%)	27 (73.0%)		0.95 0(0.43-2.11)	40 (60.6%)		1.57 (0.85-2.92)
**Race**							
Caucasian	120 (92.3%)	33 (89.2%)	0.580		63 (93.9%)	0.656	
Non-Caucasian	10 (7.7%)	4 (10.8%)		1.41 (0.42-4.78)	4 (6.1%)		0.76 (0.23-2.53)
**Weight**							
Mean (sd)	61.5 (11.3)	64.1 (10.8)	0.454		61.1 (12.2)	0.977	
**Chronic Diseases**							
No	63 (48.5%)	10 (27.0%)	**0.018**		26 (39.4%)	0.189	
Yes	67 (51.5%)	27 (73.0%)		2.63 (1.18-5.86)	40 (60.6%)		1.48 (0.81-2.70)
**Smoking habits**							
No	79 (60.8%)	30 (81.1%)	**0.026**		52 (78.8%)	**0.013**	
Yes	51 (39.2%)	7 (18.9%)		0.36 (0.15-0.88)	14 (21.2%)		0.42 (0.21-0.83)
**Alcohol intake**							
No	85 (65.4%)	28 (75.7%)	**0.241**		54 (81.8%)	**0.019**	
Yes	45 (34.6%)	9 (24.3%)		0.61 (0.26-1.39)	12 (18.2%)		0.42 (0.20-0.86)
**Other medication**							
< 3 drugs	114 (87.7%)	26 (70.37%)	**0.014**		50 (75.8%)	**0.036**	
≥ 3 drugs	16 (12.3%)	11 (29.7%)		3.01 (1.25-7.25)	16 (24.2%)		2.28 (1-06-4.92)
**Other hepatotoxic drugs**							
No	106 (81.5%)	21 (56.8%)	**0.003**		36 (54.5%)	**<0.001**	
Yes	24 (18.5%)	16 (43.2%)		3.36 (1.53-7.39)	30 (45.5%)		3.68 (1.91-7.09)

n – number of patients; *p* – *p* value; OR -odds ratio; 95% IC - confidence interval; sd – standard deviation. Statistically significant results are highlighted in bold.

Supplement Table S3 - Characterization of genetic risk factors in mild hepatitis and DILI

Genotypes	Controls n (%)	Controls vs. Mild hepatitis			Controls vs. DILI		
		n (%)	*p*	OR (95% IC)	n (%)	*p*	OR (95% IC)
***NAT2*/Acetylation status**							
SA	58 (44.6%)	26 (70.3%)	**0.007**	2.93 (1.34-6.43)	42 (63.6%)	**0.013**	2.17 (1.18-3.99)
IA+RA	65+7 (55.4%)	9+2 (29.7%)			21+3 (36.4%)		
ABCB11							
TT+TC	21+76 (74.6%)	8+13 (56.8%)	**0.038**		5+35 (60.6%)	**0.045**	
CC	33 (25.4%)	16 (43.2%)		2.24 (1.05-4.79)	26 (39.4%)		1.91 (1.01-3.59)
GSTM1							
Null	69 (53.1%)	18 (48.6%)	0.634	0.84 (0.40-1.74)	31 (47.0%)	0.419	0.78 (0.43-1.42)
Positive	61 (46.9%)	19 (51.4%)			35 (53.0%)		
GSTT1							
Null	17 (13.1%)	5 (13.5%)	0.945	1.03 (0.36-3.03)	4 (6.1%)	0.143	0.43 (0.14-1.33)
Positive	113 (86.9%)	32 (86.5%)			62 (93.9%)		
***IL6* rs1800797**							
AA+AG	16+54 (53.8%)	3+19 (59.5%)	0.545		5+35 (60.6%)	0.368	
GG	60 (46.2%)	15 (40.5%)		0.79 (0.38-1.67)	26 (39.4%)		0.76 (0.41-1.38)
***IL6* rs1800796**							
CC+CG	2+16 (13.8%)	0+6 (16.2%)	0.717		1+11 (18.2%)	0.472	
GG	112 (86.2%)	31 (83.8%)		0.83 (0.30-2.27)	54 (81.8%)		0.73 (0.32-1.61)
CYP2E1							
CC	114 (87.7%)	34 (91.9%)	0.481		59 (89.4%)	0.727	
TT+CT	1+15 (12.3%)	0+3 (8.1%)		1.59 (0.44-5.79)	0+7 (10.6%)		1.18 (0.46-3.03)

n – number of patients; *p* – *p* value; OR -odds ratio; 95% IC - confidence interval. Statistically significant results are highlighted in bold.

Supplement Table S4 - Results of logistic multivariate analysis for mild hepatitis

Variables	OR	IC 95%	*p*
Age ≥55 years	2.18	0.94 – 4.77	0.075
Other hepatotoxic drugs	**2.60**	**1.12 – 6.30**	**0.025**
Slow Acetylator	**2.39**	**1.05 – 5.44**	**0.038**
ABCB11- CC	1.91	0.84 – 4.34	0.119

OR – odds ratio; CI – confidence interval; *p* – *p* value. Nagelkerke R Square: 17.9%
